# Narrative review of data supporting alternate first-line therapies over metformin in type 2 diabetes

**DOI:** 10.1007/s40200-024-01406-6

**Published:** 2024-03-25

**Authors:** John Andraos, Shawn R. Smith, Amanda Tran, David Q. Pham

**Affiliations:** 1 Western University of Health Sciences, College of Pharmacy, Pomona, CA USA; 2 HOAG, Mary & Dick Allen Diabetes Center, Newport Beach, CA USA

**Keywords:** Metformin, Type 2 diabetes, SGLT-2 inhibitors, GLP-1 receptor agonists

## Abstract

**Purpose:**

Metformin has been the first-line treatment for type 2 diabetes mellitus as monotherapy or concomitantly with other glucose-lowering therapies due to its efficacy, safety, and affordability. Recent studies on the cardioprotective and renoprotective benefits of glucagon-like peptide-1 receptor agonists (GLP-1 RA) and sodium-glucose cotransporter-2 inhibitors (SGLT-2i) have influenced guidelines on diabetes management to consider these newer agents as alternative first-line therapies. This paper explores the literature supporting the use of these newer medications alone as a first-line agent in place of metformin.

**Methods:**

A review of citations from the most recent guidelines along with a literature search via PubMed was completed to review (1) what, historically, made metformin first-line (2) if newer agents’ benefits remain when used without metformin (3) how newer agents compare against metformin when used without it.

**Results:**

Evaluation of the historical literature was completed to summarize the key findings that support metformin as a first-line therapy agent. Additionally, an assessment of the literature reveals that the benefits of these two newer classes are independent of concomitant metformin therapy. Finally, studies have demonstrated that these newer agents can be either non-inferior or sometimes superior to metformin when used as monotherapy.

**Conclusion:**

GLP-1 RA and SGLT-2i can be considered as first line monotherapies for select patients with high cardiovascular risks, renal disease, or weight loss requirements. However, pharmacoeconomic considerations along with lesser long-term safety outcomes should limit these agents’ use in certain patients as the management of diabetes continues to transition towards shared-decision making.

**Supplementary Information:**

The online version contains supplementary material available at 10.1007/s40200-024-01406-6.

## Introduction

The International Diabetes Foundation recommended metformin as the gold standard first-line therapy for the treatment of type 2 diabetes (DMII) in 2005 due to its proven efficacy and safety profile [1]. In 2006, the American Diabetes Association/European Association for the Study of Diabetes (ADA/EASD) also endorsed metformin as the first line medication in DMII [2]. Metformin has been shown to reduce blood glucose levels, improve insulin sensitivity, reduce the risk of microvascular complications, and improve cardiovascular outcomes independent of glucose control [3].

However, in a paradigm shift reflecting recent clinical data, the guidelines for management of DMII have been modified to elevate the use of GLP-1 RA and SGLT-2i. The American Association of Clinical Endocrinology (AACE) and European Society of Cardiology in collaboration with the European Association for the Study of Diabetes (ESC/EASD) both updated their treatment algorithms in 2019 to recommend the use of GLP-1 RA and SGLT-2i as alternate therapy options to metformin based on cardiovascular (CV) and renal comorbidities independent of glycemic control [4, 5]. This was followed by the ADA updating their 2021 guidelines with the same recommendation [6]. In 2022, the ADA/EASD published a consensus report that removed metformin as the only first-line therapy and provided the emphasis of using GLP-1 RA or SGLT-2i as first line options in patients with cardiovascular and renal disease [7].

Since then, the ADA guideline of 2023 has modified its pharmacological treatment algorithm based on cardiorenal risk reduction in high-risk patients or achievement and maintenance of glycemic and weight management goals. Thus, the first-line therapy option is highly dependent on patient specific factors [8]. The AACE guideline of 2023 has included therapy options of either a GLP-1 RA or pioglitazone for patients who have experienced strokes or transient ischemic attacks, and continues to recommend mono, dual, or triple therapy based on HbA1c laboratory values [4, 9]. The ESC/EASD has not released an updated guideline for the management of pre-diabetes, diabetes, and cardiovascular disease.

GLP-1 RA offer a wide range of benefits in the management of DMII. Clinical trials have demonstrated their efficacy in improving glycemic control by stimulating glucose-dependent insulin secretion and suppressing glucagon release [10]. Additionally, GLP-1 RA can help patients achieve significant weight loss due to their effects on appetite suppression, delayed gastric emptying, and promoting satiety which can be beneficial for patients with obesity [11]. Other studies have demonstrated that GLP-1 RA have large CV benefits such as reduction in major adverse CV events [11]. GLP-1 RA have proven to be a favorable therapeutic option in the management of DMII.

SGLT-2i are also a valuable therapeutic class for treatment of DMII for similar reasons as GLP-1 RA. These agents improve glycemic control by inhibiting renal glucose reabsorption, resulting in increased urinary glucose excretion [12]. SGLT-2i can provide reductions in body weight and blood pressure, which offer advantages for patients with T2DM with obesity or hypertension [13]. SGLT-2i have shown CV benefits as evidenced by reduced rates of cardiovascular deaths and hospitalizations from heart failure in clinical trials [14]. These agents also exhibit renoprotective effects and have been shown to slow the progression of diabetic kidney disease [15]. The multifaceted benefits of SGLT-2i position them as valuable therapeutic options in the comprehensive management of DMII.

By thoroughly examining supportive literature, this review article aims to assess whether metformin should remain a solitary first-line agent or if clinicians may consider GLP-1 RA and SGLT-2i as first line therapy options without metformin on board.

### Metformin-efficacy and safety

Metformin was first introduced to the US market in 1995. Soon after FDA approval, Defronzo et al. published a study with metformin as monotherapy and found the agent decreased mean HbA1c by 1.3% compared to a 0.4% increase in the placebo group after 29 weeks [16]. In 1998, the United Kingdom Prospective Diabetes Study (UKPDS) not only found a greater improvement in glycemic control in patients taking metformin compared with the conventional treatment arm, but also showed that metformin therapy resulted in a reduction in hypoglycemic events and weight gain compared with sulfonylureas and insulin. Although data from UKPDS were published more than 2 decades ago, metformin was the first glucose-lowering agent associated with improved cardiovascular (CV) outcomes in a randomized trial [3]. The greater availability of trial data for the newer glucose-lowering agents and change in trial design complicate therapeutic profile comparisons of these agents and metformin.

In UKPDS, individuals eligible for randomization had newly diagnosed type 2 diabetes with fasting plasma glucose greater than 109.8 mg/dL and less than 270 mg/dL after a 3-month run-in period. Patients with active CV disease were excluded unlike many recent studies that compare CV outcomes which often focus on these patients. Randomization was stratified partially by weight: in 15 of the 23 centers, 1709 overweight patients were randomized to receive open-label metformin (*n* = 342), conventional treatment with diet modifications (*n* = 411), or intensive glycemic management with a sulfonylurea or insulin (*n* = 961). Patients had a mean HbA1c of 7.2% at baseline in this newly diagnosed population, which is low compared to most current trials in more long-standing type 2 diabetes, and a mean BMI of 31.4 kg/ms2 at baseline [3]. Participants were followed for a median of 10.7 years for primary endpoints that included any diabetes-related endpoint, diabetes-related death, all-cause death. Secondary endpoints were myocardial infarction, stroke, peripheral artery disease, and microvascular disease. Randomization to metformin was associated with reduced risk of any diabetes-related endpoint (HR 0.68, 0.53–0.87), all-cause mortality (HR 0.64, 0.45–0.91) and diabetes-related death (HR 0.58, 0.37–0.91) compared with the control group. Significant reductions were also reported for the risk of myocardial infarction (HR 0.61, 0.41–0.89). The risk of a combined macrovascular endpoint (myocardial infarction, angina, stroke and peripheral vascular disease, sudden death) was reduced by 30% in the metformin group, relative to control. UKPDS participants were followed for outcomes for a further 10 years after the end of randomized treatment [17]. The outcomes for which significant risk reductions occurred in metformin-treated patients in the randomized phase remained significantly reduced after post-trial follow-up. There was no significant effect on the incidence of stroke, peripheral vascular disease, or microvascular disease (when considered as individual outcomes) in either phase [17].

While UKPDS was conducted exclusively in newly diagnosed patients, another small, randomized trial enrolled a population with longstanding type 2 diabetes. In the HOME trial, Kooy et al. enrolled 390 insulin dependent participants with an average diabetes duration of 13 years who were randomized to receive metformin or placebo for an average follow-up of 4.3 years [18]. The primary endpoint was a composite of three microvascular and multiple macrovascular outcomes. There was no significant difference between groups for the primary microvascular and macrovascular composite endpoint (HR 0.92; 0.72–1.18) or the secondary composite microvascular endpoint (HR 1.04; 0.75–1.44). The secondary macrovascular composite endpoint was reduced significantly by metformin versus placebo (HR 0.61; 0.40–0.94). In a mediation analysis, this was partly explained by a mean reduction of 3.07 kg bodyweight. Overall, metformin added to insulin in patients with T2DM improved body weight, glycemic control, and insulin requirements and reduced secondary macrovascular composite endpoints after a follow-up period of 4.3 years [18].

Additionally, metformin (*n* = 1,454) was compared to glyburide (*n* = 1,441) and rosiglitazone (*n* = 1,456) as monotherapy in patients with type 2 diabetes in the A Diabetes Outcome Progression Trial (ADOPT) [19]. At 1 year, HbA1c reductions were equivalent in all treatment arms. By the end of the 5 years, HbA1c reduction was lowest in patients treated with rosiglitazone, intermediate in those treated with metformin and highest in those treated with glyburide [19]. Though this was not a prospective outcomes trial, it is important to note that the primary outcome was a measure of glycemic durability, with CV events (MI, stroke, or heart failure) recorded as adverse events rather than being listed as pre specified outcomes. In this trial, 62 CV events occurred in the rosiglitazone group, 58 in the metformin group and 41 in the glyburide group. During this time, concerns for adverse events such as edema, weight gain, heart failure, and fractures led to a significant decrease in use of thiazolidinediones and therefore leading to the increased use of metformin [19].

In 2008, the US Food and Drug Administration (FDA) introduced new guidelines for the CV evaluation of glucose-lowering treatments which was prompted initially by concern over drug safety following the publication in 2007 of a meta-analysis of trials of a thiazolidinedione glucose-lowering agent, rosiglitazone. Data suggested a significant increase in the risk of mortality by 43% (HR 1.43;1.03–1.98) and a concern trending towards an increase in the risk of CV death (HR 1.64; 0.98–2.74) for rosiglitazone relative to other comparators [20]. These findings were ultimately refuted in 2013 but the literature led to increased CV safety oversight within the development of new therapies for type 2 diabetes [21, 22]. Before 2008, the evaluation of CVD outcomes in diabetes was performed rarely, and individual trials were designed without specific reference to the design of other trials. After 2008, there have been numerous randomized, placebo-controlled, outcome trials with primary MACE outcomes [23]. More recently, a 3-year, randomized clinical trial conducted in China compared the effects of metformin and the SU, glipizide, on CVD outcomes in 304 individuals with type 2 diabetes and pre-existing coronary artery disease which included documented prior MI or stenosis of ≥ 50% in a major coronary artery [24]. Average diabetes duration was ~ 6 years and 9% were receiving insulin. The primary outcome of a composite endpoint of CV death, all-cause death, non-fatal MI, non-fatal stroke, or arterial revascularization was reduced in the metformin group relative to glipizide (HR 0.54; 0.30–0.90) [24].

Metformin has also been compared as monotherapy with exenatide once weekly, pioglitazone and sitagliptin in the DURATION-4 study [25]. Though exenatide may be less potent than other GLP-1 RA, at 26 weeks, HbA1c was reduced by 1.53% with exenatide from a baseline A1c of 8.4% and 1.48% with metformin from a baseline A1c of 8.6%. There were similar A1c reductions for the groups with pioglitazone (1.63%) and with sitagliptin (1.15%). Exenatide and metformin were both associated with a 2 kg weight loss while the sitagliptin group had a loss of 0.8 kg. The pioglitazone group was associated with a 1.6 kg weight gain. Thus, as monotherapy, metformin has equivalent or better efficacy compared with thiazolidinediones, a sulfonylurea, a long-acting GLP-1 receptor agonist, and a DPP-IV inhibitor in the short term (26–52 weeks) and is comparable to a TZD and superior to a sulfonylurea in the long term (5 years) [25]. Rates of hypoglycemia were low with metformin and comparable to those seen with the thiazolidinediones, exenatide and sitagliptin. Observed rates of hypoglycemia were significantly lower than sulfonylureas in these studies. Although nausea was more common with metformin than with the TZDs, glyburide, or sitagliptin, it was less common than with exenatide [25].

A Comparative Effectiveness Study randomized 5,047 type 2 diabetes patients to glimepiride, sitagliptin, liraglutide or insulin glargine, added to existing metformin therapy. Study participants had to be diagnosed within the previous 10 years and treated with at least 500 mg of metformin per day, but no other glucose-lowering medications, and a glycated hemoglobin level of 6.8 to 8.5% without history of heart failure or CVD [26]. While the primary outcome relates to glycemic control, CV events and mortality are included as secondary outcome measures [27]. Hypertension (67%) and hyperlipidemia (72%) were prevalent at baseline, and the majority were on antihypertensive (69%) and lipid lowering (66%) medications. After a mean follow-up duration of 5 years, the treatment groups did not differ with respect to MACE, hospitalization for heart failure, death from cardiovascular causes, or all deaths. When one treatment was compared with the combined results of the other three treatments, the hazard ratios for any cardiovascular disease were 1.1 (0.9–1.3) in the glargine group, 1.1 (0.9–1.4) in the glimepiride group, 0.7 (0.6–0.9) in the liraglutide group, and 1.2 (1.0-1.5) in the sitagliptin group. While the trial was not powered as a cardiovascular outcomes trial, results confirm the cardiovascular safety of common agents when used alongside metformin [27].

Meta-analyses regarding metformin’s effects on adverse CV outcomes provide conflicting conclusions [28–30]. Limited availability of long-term evaluations of metformin hinders effective meta-analysis. For example, one recent meta-analysis of the effect of metformin on the incidence of MI included seven trials, with durations ranging from 6 months to 10 years, and with numbers of events ranging from 14 to 423 [28]. In 2019, a very large meta-analysis included more than 1 million patients, who participated in 40 randomized or observational evaluations of metformin [31]. Treatment with metformin versus no metformin therapy was associated with reduced risk of CV death (HR 0.81; 0.79–0.84). Moreover, all-cause mortality was reduced in the overall population (HR 0.67; 0.60–0.75) in those with prior MI (HR 0.79; 0.68–0.92) and in those with prior congestive heart failure (HR 0.84; 0.81–0.87). The frequency of CV events was also reduced although no significant effect was observed in the absence of type 2 diabetes [31].

Overall, the randomized trials that evaluated metformin were small by current trial standards, although the 10-year UKPDS employed a considerably longer follow-up duration than most other studies. The Kaplan–Meier curves for macrovascular outcomes in the metformin and conventional therapy groups did not diverge until about 6 years into the study [3]. Larger studies which were not designed as outcomes trials were of shorter duration and insufficient to show benefit. The ongoing VA-IMPACT outcomes trial in subjects with prediabetes will provide additional clinical data to determine the cardiovascular efficacy of metformin. Results are expected to be reported in mid-2024. However, from approval until recently, metformin has shown at least non-inferiority and many times superiority in glycemic, weight, cardiovascular, and adverse effect outcomes compared to older agents used for diabetes. These studies ultimately led to major guidelines historically recommending metformin as their first line agent.

### Benefits of GLP-1 RA and SGLT2i

Research has shown that GLP-1 RA and SGLT-2i in combination with metformin are effective in lowering A1c, reducing cardiovascular events, preventing renal disease progression, and decreasing body weight. Key literature is summarized in Tables 2 and 3 to illustrate the proven benefits of these therapies. It is important to note that all of these studies were done in patients taking metformin at baseline. The benefits of these drug classes were the initial driving factor in joining metformin as a first-line agent for diabetes.

### Pharmacoeconomic considerations

Few pharmacoeconomic studies have been conducted directly comparing the cost of metformin with other antidiabetic agents in the management of type 2 diabetes. Most studies compare the cost of combination metformin therapies to other metformin containing combinations. However, one Chinese study compared the long-term economic outcome of dapagliflozin versus metformin in patients with T2DM whose diet and exercise have not provided sufficient glycemic control. The study showed that dapagliflozin was more costly and produced fewer health benefits in the simulated population model and that metformin is more cost effective in comparison [32]. GLP-1 RA are also brand name and costly. No formal studies have been done comparing GLP-1 RA and MTF.

GLP-1 RA and SGLT-2i are among the highest cost classes of medications for the treatment of diabetes. Although cost to patients will differ based on insurance, Table 1 shows the average wholesale price (AWP) of common GLP-1 RA and SGLT-2i compared to metformin. Despite GLP-1 RA and SGLT2i being covered for many patient insurances, this elevated AWP is important to note for patients with co-insurances, deductibles, the Medicare coverage gap, patients without insurance, and for the total cost of healthcare. The disparities in cost compared to metformin are likely to affect pharmacoeconomic models and must be considered when selecting agents for patients.

### Methodology of article collection

New medications such as GLP-1 RA (first approved in 2005) and SGLT-2i (first approved in 2013) have emerged, and there is growing recognition that these alternative approaches may be considered without metformin already being used concurrently. In order for these considerations to be substantiated, evidence to support the use of GLP-1 RA and/or SGLT-2i without the use of metformin is required. Do GLP-1 RA and SGLT-2i reduce CV events and provide renal benefits in patients with type 2 diabetes that are not using metformin? If so, does their consideration in individuals with existing or high risk of cardiovascular disease, heart failure, or chronic kidney disease, regardless of use with metformin make sense?

To answer these questions, literature was collected from two sources. First, a review of the ADA guideline citations helped direct the authors to assess the reasons the Association updated their guidance. Additionally, a subsequent search on PubMed was done to search for any missing information regarding the use of GLP-1 RA or SGLT-2i without metformin in June, 2023 by one author independently. Since the previous guidelines recommended most patients to take metformin as a first line agent, literature was limited. However, 6 articles were found and discussed in the following section.

Even with evidence showing the utility of GLP-1 RA and SGLT-2i without metformin, these medications should still not be used preferentially over metformin unless evidence shows these agents are superior or at least non-inferior to metformin. In order to compare the safety and efficacy of newer agents that treat diabetes alone compared to metformin, a PubMed search was conducted. The search was completed in June, 2023 independently by one author using the “AND” boolean to capture titles with both keywords “metformin” and each medication in the SGLT-2i and GLP-1 RA drug classes (Appendix 1). A total of 493 results were found. After reviewing the articles based on the pre-specified inclusion and exclusion criteria (Appendix 2), 14 articles comparing metformin to newer agents monotherapy were extracted. Four of the articles compared SGLT-2i to metformin while 10 articles compared GLP-1 RA to metformin. Full results related to glycemic control, cardiovascular disease, cardiovascular disease risk factors, and weight of the articles can be found in Table 4 but are summarized in the section titled “Metformin Alone vs. Newer Agents Alone”.

### GLP-1 RA and SGLT-2i without metformin

In 2020, Crowley et al. published a post-hoc analysis that observed the effects of liraglutide versus placebo with existing diabetes therapy on CV outcomes in patients with type 2 diabetes [33]. This study used data collected from the Liraglutide Effect and Action in Diabetes: Evaluation of Cardiovascular Outcome Results (LEADER) trial. The primary outcome was time from randomization to the first occurrence of cardiovascular death, myocardial infarction, or stroke. There were 9,340 participants in the LEADER trial and 2,242 (24%) were not using metformin at baseline. Baseline covariates were adjusted for many parameters including but not limited to age, sex, diabetes duration, A1C, other antihyperglycemic medications used, prior myocardial infarction and prior stroke. Liraglutide with metformin did not significantly reduce the primacy outcome versus placebo with metformin (HR 0.97, 0.85–1.10). However, those taking liraglutide without metformin showed a significant reduction in the primary outcome versus those taking placebo without metformin (HR 0.79, 0.64–0.97) [33]. This means that the effects of liraglutide appeared to be significantly greater in the group without metformin on board compared to when liraglutide was used in conjunction with metformin. The results seem to indicate that the CV benefits of liraglutide are independent of metformin use though it may also highlight the protective effects of metformin in the placebo group. The main limitation of this post-hoc analysis is that the LEADER trial was never explicitly designed to evaluate the effect that liraglutide might have on cardiovascular outcomes with or without metformin use. Another sufficiently powered randomized trial would be needed to observe any true differences in the efficacy of liraglutide on cardiovascular outcomes when used with or without metformin. Overall, based on this study, there appears to be evidence supporting the use of liraglutide without metformin reducing the time to first occurrence of cardiovascular death, myocardial infarction, or stroke.

Neuen et al. published a meta-analysis that studied patients with type 2 diabetes using SGLT-2i with and without metformin on outcomes related to CV function, kidney function, and all-cause mortality [34]. This meta-analysis pooled data from six randomized, placebo-controlled, clinical trials that involved 4 different SGLT-2i for a total of 51,743 participants. The effect from treatment was reported as hazards ratios and 95% confidence intervals using random-effects meta-analysis. The main outcomes studied were incidences of major adverse cardiovascular events (MACE), hospitalization for heart failure, or cardiovascular death. The use of SGLT-2i without metformin varied between 18 and 79%. The results indicated that SGLT-2i decreased the risk of MACE, with and without concomitant metformin use (HR 0.93, 0.87-1.00 and HR 0.82, 0.71–0.86, respectively; *P*-heterogeneity = 0.14). There were also clear and separate reductions in hospitalization for heart failure or cardiovascular death with SGLT-2i, irrespective of metformin use (HR 0.79, 0.73–0.86 and HR 0.74, 0.63–0.87, respectively; *P*-heterogeneity = 0.48), as well as for major kidney outcomes and all-cause mortality (all *P*-heterogeneity > 0.40) [34]. This study showcased that usage of SGLT-2i in patients with type 2 diabetes resulted in reductions in cardiovascular outcomes, kidney outcomes, as well as all-cause mortality regardless of if patients were taking metformin. The results of this meta-analysis support the new recommendations from the ADA and EASD 2022 guidelines suggesting SGLT-2i be used in patients with type 2 diabetes at high or very high cardiovascular risk, regardless of if they are receiving or not receiving metformin. As with all meta-analyses, limitations include the inability to fully consider all important covariates, bias on overstatements of the strength and precision of the results, and bias thresholds of when to include studies that may be too heterogeneous.

Perhaps the most relevant study that the ADA EASD 2022 guideline referenced was the meta-analysis conducted by Masson et al [35]. This was a study designed to evaluate the effect of SGLT-2i and GLP-1 RA on MACE specifically in patients with type 2 diabetes that were metformin-naive. The researchers analyzed randomized controlled clinical trials that focused on the effects of GLP-1 RA and SGLT-2i. They used multiple databases including PubMed/MEDLINE, Embase, and Cochrane Controlled Trials to identify relevant studies. The primary outcome investigated was incidence of MACE. Additionally, they explored a secondary outcome in a subset of studies in patients using SGLT-2i which was cardiovascular death and hospitalization for heart failure. Researchers applied a random-effects meta-analysis model to describe the data. In total, there were six eligible trials, consisting of three studies on patients receiving SGLT-2i and three trials on patients using GLP-1 RA, that involved a total of 13,049 participants. The group taking GLP-1 RA and SGLT-2i showed a significant reduction in MACE (OR 0.80, 0.70–0.93;I^2^:53%). When separated into specific drug groups, the group receiving GLP-1 RA showed a significant reduction in MACE (OR 0.77, 0.67–0.88). In contrast, the group receiving SGLT-2i did not show a significant reduction in MACE (OR 0.85, 0.63–1.15). Furthermore, the group receiving SGLT-2i showed a significant reduction in hospitalization for heart failure or cardiovascular mortality (OR 0.67, 0.47–0.95;I^2^:78%) [35]. These results indicate that GLP-1 RA independently have a beneficial effect on reducing MACE in metformin naive patients with type 2 diabetes whereas SGLT-2i do not. However, SGLT2i do appear to be associated with a reduction in cardiovascular death as well as hospitalization for heart failure. Limitations included clinical heterogeneity which included different patient characteristics, different use of other antihyperglycemic medications, and different follow up schedules.

Tsapas et al. conducted a meta-analysis that determined the effectiveness of GLP-1 RA with and without metformin on cardiovascular outcomes in patients with type 2 diabetes [36]. Four trials were included for a total of 45,456 patients. The primary outcome was the incidence of MACE. Secondary outcomes were incidence of myocardial infarction, stroke, cardiovascular death, all-cause mortality, and hospitalization for heart failure. The clinical trials included the use of albiglutide (currently discontinued in the United States), dulaglutide, exenatide once weekly, and liraglutide. Overall, the effect of GLP-1 RA regardless of metformin use on incidence of MACE was significantly lower by 13% (HR 0.87, 0.82–0.93) compared to subjects not taking GLP-1 RA at all. When this data was stratified to patients taking GLP-1 RA with metformin versus without metformin, the results on incidence of MACE were also significantly lower in both groups (HR 0.91, 0.85–0.97 and HR 0.80, 0.72–0.90) respectively [36]. The results of this study seem to indicate that using GLP-1 RA whether it be with or without metformin still resulted in a significant reduction in cardiovascular death and all-cause mortality. The use of GLP-1 RA with or without metformin also appeared to be neutral on stroke, myocardial infarction, and hospitalization from heart failure.

Another compelling meta-analysis that showcased GLP-1 RA without metformin use on cardioprotection was by Lavalle-Cobo et al [37]. This study also evaluated the incidence of MACE as the primary outcome. The secondary outcomes were incidences of all-cause mortality and cardiovascular death. Seven trials for a total of 11,510 patients were included. When GLP-1 RA were used without metformin, it resulted in a significant reduction in MACE incidence (HR 0.86, 0.79–0.94). There were no significant reductions in the secondary endpoint of all-cause mortality (HR 0.86, 0.73-1.00) or cardiovascular death (HR 0.81, 0.63–1.05) [37]. These results support the idea that GLP-1 RA are beneficial on cardiovascular outcomes independent of metformin use.

Finally, Husain et al. published a post-hoc subgroup analysis of SUSTAIN 6 and PIONEER 6 which evaluated the use of injectable and oral semaglutide without metformin on cardiovascular events [38]. Additionally, a trial-level meta-analysis was conducted using data from seven cardiovascular outcome trials with GLP1-RA: SUSTAIN 6-semaglutide SQ, PIONEER 6-semaglutide PO, HARMONY OUTCOMES-albiglutide, LEADER-liraglutide, REWIND-dulaglutide, EXSCEL-exenatide-ER, and AMPLITUDE-O-efpeglenatide. Patients with type 2 diabetes at high risk of a cardiovascular event were randomized to either GLP-1 RA or placebo in addition to standard of care. The primary endpoints were incidences of MACE, cardiovascular death, myocardial infarction, or stroke. Semaglutide SQ once weekly, semaglutide PO once daily, liraglutide, dulaglutide, exenatide once weekly, and efpeglenatide (not used in the United States) were studied. The effect from treatment was reported as hazards ratios and 95% confidence intervals using random-effects meta-analysis. SUSTAIN 6 and PIONEER 6 included 6,480 patients, of whom 1,620 (25%) were not taking metformin. There appeared to be a reduction in the risk of MACE with semaglutide versus placebo in the metformin and non-metformin subgroups (HR 0.70, 0.55 to 0.89 and HR 0.86, 0.60 to 1.22 respectively) [38]. Although semaglutide did not show a statistically significant difference in MACE in the non-metformin group, the study also concluded there was no significant interaction between the treatment effect on MACE and the use of metformin [38].

Based on these studies there appears to be mostly retrospective and some prospective evidence showing the cardiovascular benefits of GLP-1 RA and SGLT-2i regardless of metformin use.

### Metformin alone vs. newer agents alone

Among the four studies comparing metformin to SGLT-2i, two studies compared canagliflozin to metformin. The first compared the reduction of HbA1c and weight in the combination of the two medications versus either medication on its own in 1,186 patients [39]. A secondary analysis was conducted to compare canagliflozin monotherapy to metformin monotherapy. This study showed non-inferiority in the ability to reduce HbA1c with both strengths (100 mg: -0.06% and 300 mg: -0.11%) of canagliflozin compared to metformin. It also showed statistically significant weight loss with canagliflozin (100 mg: -0.9 kg, 300 mg: -1.8 kg) compared to metformin [39]. Another smaller study compared canagliflozin monotherapy to metformin and observed not only changes in HbA1c and FBG, but also markers of insulin resistance. Though no statistical analysis was done and values were somewhat similar, canagliflozin showed improvement in HbA1c (-0.6%), FBG (-0.2 mg/L), Homeostatic Model Assessment for Insulin Resistance (HOMA-IR) scores (-0.5), C-Reactive Protein (CRP) (-0.9 mmol/L), subcutaneous adipose tissue (-1.1 cm2), visceral adipose tissue (-5.0 cm2), and nitric oxide levels (+ 2.3 umol/L) compared to metformin [40]. Two studies comparing dapagliflozin to metformin showed similar results: HbA1c reductions between dapagliflozin and metformin monotherapy were non-inferior [41]. However, a 52-week study of 248 patients also showed a statistical significant difference in systolic blood pressure (-2.3 mmHg) and diastolic blood pressure (-1.4 mmHg) reduction between dapagliflozin and metformin [42]. Safety outcomes in all studies were as expected showing increased gastrointestinal adverse effects with metformin and increased genitourinary adverse effects with SGLT-2i[39-42]. No articles comparing cardiovascular, renal, or other benefits were found. The results of the aforementioned studies are also limited as most outcomes were not the primary outcome of the studies. Overall, SGLT-2i seem to be at least non-inferior at lowering HbA1c and somewhat superior at lowering risk factors of cardiovascular disease including, weight, blood pressure, and markers of insulin resistance.

Several studies also compared the effects of GLP-1 RA alone to metformin. A 52-week long study comparing 807 patients on either dulaglutide or metformin showed statistically significantly greater HbA1c lowering with the GLP-1 RA at all doses (0.75 mg: -0.15% and 1.5 mg: -0.22%) compared to metformin at 26 weeks. Secondary outcomes also showed similar weight loss with dulaglutide 1.5 mg and less weight loss with dulaglutide 0.75 mg compared to metformin at 52 weeks. A greater percentage of patient achieved A1C goal in both the dulaglutide treatment arms compared to metformin. Both GLP-1 RA doses also improved HOMA2-%B (beta cell output), HOMA2-S (insulin sensitivity), and glucagon levels compared to metformin [43].

Similar results in regard to HbA1c lowering were seen with exenatide twice daily though it is important to note the studies were all done in obese patients unlike the dulaglutide study [44, 45]. Additionally, the study showed improvement in triglyceride reduction with exenatide compared to metformin (-1.23 mmol/L) though the outcome was secondary in nature [44]. A study comparing weekly exenatide not only showed non-inferiority at reducing HbA1c compared to metformin (-1.53% vs. -1.48%), but also showed non-inferior HbA1c reduction compared to pioglitazone (-1.53% vs. -1.63%) and superior HbA1c reduction compared to sitagliptin (-1.53% vs. -1.15%) [25].

Finally, liraglutide showed slightly weaker glycemic outcomes compared to other GLP-1 RA in two studies of overweight and obese Asian patients. The studies showed only non-inferiority in glycemic outcomes but did show superiority in weight loss compared to metformin in one of the two studies. It is important to note that the studies were smaller in size comparatively and did not utilize the maximum FDA-approved dosage of liraglutide [46, 47]. Alternatively, liraglutide did show greater improvements of hepatic enzyme levels compared to gliclazide, a sulfonylurea, and similar improvements of hepatic enzyme levels compared to metformin [48].

Though no studies compared the cardiovascular outcomes of metformin versus GLP-1 RA, exenatide BID showed similar reactive hyperemia index reductions, a measure of endothelial function compared to metformin [49]. Liraglutide also showed improvements in cardiovascular risk factors compared to metformin including BMI [-0.34 kg/m2 (*p* < 0.01)], SBP [-7 mmHg (*p* < 0.001)], DBP [-3 mmHg (*p* < 0.001)], total cholesterol [-0.2 mmol/L (*p* = 0.033)], LDL-C [-0.2 mmol/L (*p* = 0.033)], CRP [-1.9 mg/L (*p* < 0.001)], LVEDD [-4 mm (*p* < 0.001)], EF [+ 2% (*p* = 0.004)], E/A ratio [+ 0.11 (*p* < 0.001)] [47]. These outcomes suggest that GLP-1 RA alone may have cardiovascular benefits compared to metformin alone though it is important to note that these outcomes were all surrogates.

Safety outcomes were consistent with previous literature for both metformin and GLP-1 RA. Though a known effect, a subpopulation analysis of KIND-LM explored the effect of GLP-1 RA on pancreatic enzymes. The study showed modest but statistically significant increases in amylase and lipase levels in the liraglutide group compared to the metformin group though none of these patients developed pancreatitis [50]. The correlation between GLP-1 RA and increases in pancreatic enzymes are known and should be considered when selecting agents to treat diabetes.

Comparative studies between GLP-1 RA or SGLT-2i and metformin are limited as older guidelines recommended almost all patients to be on metformin. This limits the newer medications’ manufacturer’s ability to have strong prospective studies. Similarly, as metformin is now generic, few researchers have incentive to create strong research supporting metformin’s use. Though there is a brevity of strong literature supporting the use of newer agents for diabetes prior to initiating metformin, the available evidence shows that newer agents are at least non-inferior and, in some cases, superior to metformin at lowering glucose when used alone. GLP-1 RA and SGLT-2i also have additional benefits such as improvements in weight, blood pressure, insulin sensitivity, and other risk factors of cardiovascular disease when used alone compared to when metformin is used alone. With the update in the ADA guidelines, stronger comparative literature from GLP-1 RA and SGLT-2i manufacturers will likely follow, including potential comparisons of cardiovascular outcomes and pharmacoeconomic considerations.

### Conclusion and future studies

Metformin has been used as first-line therapy for the treatment of DMII for nearly over two decades given data that supports improved cardiovascular outcomes, reduction in HbA1c, weight neutrality, and overall safety profile. Over the years, numerous studies have outlined the cardiovascular and renal benefits of GLP-1 RA and SGLT-2i in combination with metformin. Additional benefits from these studies are low risk of hypoglycemia and significant weight loss. Several post-hoc analyses and meta-analyses illustrate that GLP-1 RA and SGLT-2i can provide similar benefits without metformin. However, to conclude that SGLT-2i and GLP-1 RA can be used preferentially over metformin would require head-to-head trials to determine whether these newer agents are superior to metformin.

Although not as thoroughly studied and assessed, GLP-1 RA and SGLT-2i compared to metformin may have potential benefit in decreasing insulin resistance and improving beta cell output. Further studies need to be completed to assess the clinical significance of how these two agents may improve beta cell output, but it would be highly beneficial for patients with type 2 diabetes to preserve beta cell function to maintain glucose homeostasis. One limitation to this review article is the lack of bias assessment. The authors aimed to include all article relevant to allow readers to make their own conclusions based on all of the information. Though the authors made their own conclusions based on collected articles, readers must recall that the strength of the conclusions are only as strong as the data in these articles.

The guidelines for management of DMII have been revised to recommend first line therapy options that can provide improved cardiorenal and weight outcomes compared to previous guidelines that focused more on glycemic control. The ADA 2023 guideline stratified its treatment algorithm to recommend either a GLP-1 RA or SGLT-2i for individuals identified to have high cardiovascular and renal risks. The inclusion criteria used to identify this high-risk group encompasses a large proportion of patients with DMII. This showcases how the ADA guideline may be emphasizing GLP-1 RA and SGLT-2i as treatment options over metformin; however, treatment options are still limited by patient specific factors and the cost of therapy. Compared to SGLT-2i and GLP-1 RA, metformin is more affordable which may impact medication adherence and glycemic outcomes. Further pharmacoeconomic studies may be needed to assess how often GLP-1 RA and SGLT-2i can be used. This, in fact, has caused many insurance companies to introduce step-therapies for newer agents limiting the access to patients not taking metformin. Metformin has also been shown to be safe and tolerable while the long-term side effects of SGT-2i and GLP-1 RA have yet to be fully assessed in the current literature. In summary, although these newer agents are a good option for some patients with cardiovascular and renal disease, practitioners should not completely disregard metformin as a first-line option especially since the comparative cardiovascular outcomes (which are the major highlighted benefit of the medications) are limited and mostly focus on surrogate endpoints.

As newer agents begin having more positive cardiovascular outcome trials, changes in guidelines will follow suit. Similar to metformin no longer being the only first line agent for treatment of DMII, other guidelines such as the cholesterol guidelines may follow as manufacturers of brand name agents push for the support of their medications. Review of the literature indicates that metformin can continue to be considered as first-line therapy for the treatment of DMII. The data demonstrates the efficacy and safety of GLP-1 agonists and SGLT-2 inhibitors as monotherapy, which may allow them to be considered as first-line therapies based on patient specific factors such as cardiorenal benefits, weight reduction, and cost of medication.

### Electronic supplementary material

Below is the link to the electronic supplementary material.

Supplementary Material 1

Supplementary Material 2
