# Supplementary material for: Narrative review of data supporting alternate first-line therapies over metformin in type 2 diabetes
Source: J Diabetes Metab Disord. 2024 Mar 25;23(1):385–94. doi: 10.1007/s40200-024-01406-6 (PMC11196467; doi:10.1007/s40200-024-01406-6)
Supplement: Supplementary file 1 — Supplementary Material 1 [file 40200_2024_1406_MOESM1_ESM.docx]

**Table 1: Average Wholesale Price (AWP) of GLP-1 RA and SGLT-2i Compared to Metformin**

| **Medication** | **AWP (28-day supply)** |
| --- | --- |
| Dulaglutide SC (GLP-1 RA) | $1,117.04 |
| Semaglutide SC (GLP-1 RA) | $1,497.24 |
| Semaglutide PO (GLP-1 RA) | $1,048.04 |
| Canagliflozin (SGLT-2i) | $670.32 |
| Dapagliflozin (SGLT-2i) | $633.08 |
| Empagliflozin (SGLT-2i) | $664.44 |
| Ertugliflozin (SGLT-2i) | $381.64 |
| Metformin (biguanide) | $2.24 |

Table 1 shows the comparative average wholesale prices of commonly used GLP-1 RA and SGLT-2i compared to metformin.

**Table 2: Effects of GLP-1 RA with Metformin**

| **TRIAL** | **SUSTAIN 6** | **SUSTAIN 7** | **PIONEER 6** | **PIONEER 3** | **REWIND** | **AWARD-6** | **LEADER** |
| --- | --- | --- | --- | --- | --- | --- | --- |
| Medication | Subcutaneous semaglutide vs placebo | Subcutaneous semaglutide vs dulaglutide | Oral semaglutide vs placebo | Oral semaglutide vs sitagliptin | Dulaglutide vs placebo | Dulaglutide vs liraglutide | Liraglutide |
| Median duration of follow-up (years) | 2.1 | 0.125 | 1.3 | 1.5 | 5.4 | 0.08 | 3.8 |
| Patient (n) | 3,297 | 1,201 | 3,183 | 1,864 | 9,901 | 599 | 9,340 |
| Metformin at baseline (%) | 74 | 100 | 77 | 100 | 81 | 100 | 76 |
| Cardiovascular Outcome | 6.6% vs 8.9% (HR 0.74; 0.58 to 0.95)^a^ | -- | 3.8% vs 4.8% (HR 0.79;0.57to 1.11)^a^ | -- | 12% vs 13.4% (HR 0.88; 0.79 to 0.99)^b^ | -- | 13% vs 14.9% (HR 0.87; 0.78 to 0.97)^a^ |
| A1c reduction | Semaglutide 0.5 mg:  -1.1% vs 0.4% (p<0.001)  Semaglutide 1 mg:  -1.4% vs 0.4% (p<0.001) | Semaglutide 0.5 mg vs dulaglutide 0.75 mg:  -1.5% vs 1.1% (p<0.0001)  Semaglutide 1 mg vs dulaglutide 1.5 mg:  -1.8% vs -1.4% (p<0.0001) | -1.0% vs -0.3% | Semaglutide 7/14 mg vs sitagliptin 100 mg:  -0.3%/-0.5% vs -0.4% (p<0.001 for both) | -0.61 (p<0.0001) | Dulaglutide 1.5 mg vs liraglutide 1.8 mg:  -1.42% vs -1.36% (p<0.0001) | -0.30+0.28 vs -0.06+0.30 (p<0.001) |
| Hypoglycemia | Semaglutide 0.5 mg:  23.1% vs 21.5%  Semaglutide 1 mg:  21.7% vs 21% | Semaglutide vs dulaglutide:  1% vs 2% | 1.4% vs 0.8% | -- | 1.3% vs 1.5% (p=0.38) | 9% vs 6% | 1.3% vs 1.0% |
| Weight loss | -- | Semaglutide 0.5 mg vs dulaglutide 0.75 mg:  -4.6% vs -2.3% (p<0.0001)  Semaglutide 1 mg vs dulaglutide 1.5 mg:  -6.5% vs -3.0% (p<0.0001) | -4.2 kg vs -0.8kg | -1.6 kg/-2.5 kg vs -0.6 kg (p<0.001 for both) | -- | -2.9 kg vs -3.61 kg (p<0.011) | -8.4+7.3 kg vs -2.8+6.5 kg (p<0.001) |

^a^Composite primary outcome of cardiovascular mortality, nonfatal MI, or nonfatal stroke (MACE)

^b^Composite primary outcome of vascular death, nonfatal MI, or nonfatal stroke

Table 3 of SGLT-2 with metformin and A1c, CVD/renal, hypo, weight loss

Table 2 summarizes key studies showing the Glycemic, Cardiorenal, Hypoglycemic, and Weight Loss effects of GLP-1 RA along with metformin.

**Table 3: Effects of SGLT-2i with Metformin**

| **TRIAL** | **EMPA-REG/EMPA-REG OUTCOME (ESRD)** | **CANVAS/CANVAS-R** | **DECLARE-TIMI** | **VERTIS CV** |
| --- | --- | --- | --- | --- |
| Medication | Empagliflozin vs placebo | Canagliflozin vs placebo | Dapagliflozin vs placebo | Ertugliflozin vs placebo |
| Median duration of follow-up (years) | 3.1 | 3.6 | 4.2 | 3.5 |
| Patient (n) | 7,020 | 10,142 | 17,160 | 8,246 |
| Metformin at baseline (%) | 74 | 77 | 82 | 76 |
| Cardiovascular Outcomes | 12.8% vs 14.3% (HR 0.89; 0.78 to 1.01)^a^ | 26.9 vs 31.5 per 1,000 patient years (HR 0.86; 0.75 to 0.97)^a^ | MACE:  8.8% vs 9.4% (HR 0.93; 0.84 to 1.03)^a^    Cardiovascular death or hospitalization for heart failure:  4.9% vs 5.8% (HR 0.83; 0.73 to 0.95) | 11.9% vs 11.9% (HR 0.97; 0.85 to 1.11) |
| Renal Outcomes | Progression to macroalbuminuria:  11.2% vs 16.2% (HR 0.62;0.54 to 0.72)    Initiation of renal-replacement therapy:  0.6% vs 0.3% (HR 0.45; 0.21-0.97) | Progression of albuminuria:  89.4 vs 128.7 per 1,000 patient years (HR 0.73; 0.67 to 0.79)    40% reduction of eGFR, renal-replacement therapy, or renal death:  5.5 vs 9.0 per 1,000 patient years (HR 0.60; 0.47 to 0.77) | >40% decrease in eGFR to <60 ml/min/1.73m2, ESRD, or death from renal or cardiovascular cause: 4.3% vs 5.6% (HR 0.76; 0.67 to 0.87) | Death from renal causes, renal replacement therapy, or doubling of serum creatinine:  3.2% vs 3.9% (HR 0.81; 0.63 to 1.04) |
| A1c reduction | At week 206:  Empagliflozin 10 mg:  -0.24%  Empagliflozin 25 mg:  -0.36% | -0.58% (p<0.001) | 0.42% | Ertugliflozin 5 mg:  0.70%  Ertugliflozin 15 mg:  0.72% |
| Hypoglycemia | Not significant | Not significant | 0.7% vs 1.0% | Not significant |
| Weight loss | 2 kg loss for both doses | -1.60 kg | 1.8 kg | Ertugliflozin 5 mg:  2.4+3.9 kg  Ertugliflozin 15 mg:  2.8+4.0 kg |

^a^Composite primary outcome of CV mortality, nonfatal MI, or nonfatal stroke (MACE)

Table 3 summarizes key studies showing the Glycemic, Cardiorenal, Hypoglycemic, and Weight Loss effects of SGLT-2i along with metformin

**Table 4: PubMed Search Results Comparing GLP-1 RA and SGLT-2i to Metformin**

| **Search term** | **# of Results** | **Studies Meeting Criteria** | **Treatment** | **Comparator** | **Type of Study** | **N** | **Duration** | **Statistical Analyses** | **Outcome** | **Primary Outcome?** | **Result** |
| --- | --- | --- | --- | --- | --- | --- | --- | --- | --- | --- | --- |
| "semaglutide" | 21 | N/A | N/A | N/A | N/A | N/A | N/A | N/A | N/A | N/A | N/A |
| "liraglutide" | 93 | [Feng et al](https://pubmed.ncbi.nlm.nih.gov/29957886/) | Liraglutide 1.8 mg | MTF alone, gliclazide alone | Prospective | 85 | 24 weeks | Superiority | A1C  Wt  AST  ALT | Y | +0.03% (p>0.05) (vs MTF)  -0.41% (p<0.01)(vs glic)  -2 kg(p>0.05) (vs MTF)  -5.01 kg (p<0.01) (vs glic)  +4.25U/L(p>0.05) (vs MTF)  -3.46 U/L (P>0.05) (vs glic)  +0.26U/L(p>0.05)(vs MTF);  -12.03U/L (p>0.05) (vs glic) |
|  |  | [Liu et al](https://pubmed.ncbi.nlm.nih.gov/29197387/) | Liraglutide 1.2 mg (phase 1) | MTF alone (phase 1) | Prospective | 120 | 24 weeks (for phase 1) | Superiority | FBG  PPG  A1C  BMI  SBP  DBP  TG  TC  LDL-C  CRP  LVEDD  EF  E/A ratio | Y | -1 mmol/L  -0.3 mmol/L  -0.1%  -0.34 kg/m2 (p<0.01)  -7 mmHg (p<0.001)  -3 mmHg (p<0.001)  -0.1 mmol/L  -0.2 mmol/L (p=0.033)  -0.2 mmol/L (p=0.033)  -1.9 mg/L (p<0.001)  -4 mm (p<0.001)  +2% (p=0.004)  +0.11 (p<0.001) |
|  |  | [Tanaka et al](https://pubmed.ncbi.nlm.nih.gov/25739726/) | Liraglutide 0.9 mg | MTF alone | Prospective | 46 | 24 weeks | Superiority | A1C | Y | -0.95% vs -0.8% (p=0.77) |
|  |  | [Tanaka et al](https://pubmed.ncbi.nlm.nih.gov/26369653/) | Liraglutide 0.9 mg | MTF alone | Sub-group analysis | 20 | 24 weeks | Inferiority | Amylase, Lipase | N | Slight increases in amylase and lipase in liraglutide but not MTF arms |
| "dulaglutide" | 16 | [Umpierrez et al](https://pubmed.ncbi.nlm.nih.gov/24842985/) | Dulaglutide 0.75 mg, 1.5 mg | MTF alone | Prospective | 807 | 26 weeks (primary endpoint) | Non-inferiority (margin: 0.4%) with superiority test if non-inferiority is met | Change in A1C (0.75 mg)  Change in A1C (1.5 mg)% of patients with A1C <7.0% (0.75 mg), % of patients with A1C <7.0 (1.5 mg), % of patients with A1C <6.5% (0.75 mg), % of patients with A1C <6.5 (1.5 mg), Change in Weight @52 wks (0.75 mg), Change in weight @52 wks (1.5 mg) | Y | -0.15% (p<0.025 for superiority), -0.22% (p<0.025 for superiority), +8% (p=0.02 for superiority), +9% (p=0.02 for superiority), +16% (p<0.001 for superiority), +10% (0.011 for superiority), -0.07 kg (no statistical test done), +.86 kg (no statistical test done) |
| "exenatide" | 84 | [Liu et al](https://pubmed.ncbi.nlm.nih.gov/29358950/) | Exenatide 10 ug BID | MTF alone | Prospective | 230 | 12 weeks | Superiority | A1C reduction  A1C <7%  Weight | Y | -1.37% (p<0.05)  +15.5% (p<0.05)  -3.49 kg (p<0.05) |
|  |  | [Hu et al](https://pubmed.ncbi.nlm.nih.gov/29754323/) | Exenatide 10 ug BID | MTF alone | Case-control (retrospective) | 90 | 12 weeks | Superiority | Reactive hyperemia index | Y | -0.31 vs -0.14 (p<0.05 for non-inferiority) |
|  |  | [Russell-Jones et al](https://pubmed.ncbi.nlm.nih.gov/22210563/) | Exenatide 2.0 mg weekly | MTF alone, pioglitazone alone, sitagliptin alone | Prospective | 820 | 26 weeks | Non-inferiority (margin: 0.3%) with superiority test if non-inferiority is met | Change in A1C | Y | -0.05% (p=0.620 for superiority vs MTF)  +0.10 (p=0.328 for superiority vs pioglitazone)  -0.038% (p<0.01 for superiority vs sitagliptin) |
|  |  | [Yuan et al](https://pubmed.ncbi.nlm.nih.gov/22931974/) | Exenatide 10 ug BID | MTF alone | Prospective | 59 | 26 weeks | Superiority | OGTT  FBG  A1C <7%  A1C<6.5%  Weight | Y | -0.44% (p<0.05)  -0.2 mmol/L (p>0.05)  4% (p>0.05)  6% (p>0.05)  -1.99 kg (p,0.05) |
|  |  | [Dore et al](https://pubmed.ncbi.nlm.nih.gov/19278373/) | Exenatide or sitagliptin | MTF alone | ADR surveilance system | database | 156 weeks | ADR surveilance | Pancreatitis | Y | increased risk of pancreatitis with exenatide/sitagliptin |
| "albiglutide" | 4 | N/A | N/A | N/A | N/A | N/A | N/A | N/A | N/A | N/A | N/A |
| lixisenatide | 20 | N/A | N/A | N/A | N/A | N/A | N/A | N/A | N/A | N/A | N/A |
| "tirzepatide" | 1 | N/A | N/A | N/A | N/A | N/A | N/A | N/A | N/A | N/A | N/A |
| "empagliflozin" | 78 | N/A | N/A | N/A | N/A | N/A | N/A | N/A | N/A | N/A | N/A |
| "canagliflozin" | 58 | [Hao et al](https://pubmed.ncbi.nlm.nih.gov/35144596/) | Canagliflozin 100 mg | MTF alone | Prospective | 142 | 12 weeks | Superiority | HOMA-IR subq adipose tissue  visceral adipose tissue  FBG  A1C  CRP  NO | Y | -0.5  -1.1cm^2  -5.0cm^2  -0.9 mmol/L  -0.6%  -0.2 mg/L  +2.3 umol/L (no statistical significance) |
|  |  | [Rosenstock et al](https://pubmed.ncbi.nlm.nih.gov/26786577/) | Canagliflozin 100 mg, 300 mg | MTF alone | Prospective | 1186 | 26 weeks | A1C: Non-inferiority (margin: 0.35%), Wt loss: superiority | Change in A1C (100 mg)  Change in A1C (300 mg)  Wt loss (100 mg)  Wt loss (300 mg) | N | -0.06%  -0.11%  -0.9kg  -1.8 kg (all p<.05) |
| "dapagliflozin" | 96 | [Cheng et al](https://pubmed.ncbi.nlm.nih.gov/34930986/) | Dapagliflozin 10 mg | MTF alone | Prospective | 248 | 52 weeks | Superiority | A1C, FBG, fasting insulin level, HOMA-IR, weight, BMI, waist circumfurence, SBP, DBP, LDL-C, HLD-C, triglycerides | Y | 0.0%, -0.2mmol/L, +0.2 mIU/L, -0.1, 0.1 kg, 0.0 kg/m2, 0.1 cm, -2.3 mmHg (P<0.05), -1.4 mmHg (p<0.05), +.01 mmol/L, +0.01 mmol/L, +0.17 mmol/L |
|  |  | [Henry et al](https://pubmed.ncbi.nlm.nih.gov/22413962/) | Dapagliflozin 10 mg | MTF alone | Prospective | Unknown | 24 weeks | Non-inferiority (margin: unknown) | Change in A1C | N | -0.01% (p<0.5%)) |
| "bexagliflozin" | 3 | N/A | N/A | N/A | N/A | N/A | N/A | N/A | N/A | N/A | N/A |
| "ertugliflozin" | 19 | N/A | N/A | N/A | N/A | N/A | N/A | N/A | N/A | N/A | N/A |

Table 4 summarizes the PubMed search results comparing GLP-1 RA and SGLT-2i to metformin.

**Appendix 1: Pubmed Search Keywords**

| “Metformin” AND “semaglutide”  “Metformin” AND “liraglutide”  “Metformin” AND “dulaglutide”  “Metformin” AND “exenatide”  “Metformin” AND “albiglutide”  “Metformin” AND “lixisenatide”  “Metformin” AND “tirzepatide”  “Metformin” AND “empagliflozin”  “Metformin” AND “canagliflozin”  “Metformin” AND “dapagliflozin”  “Metformin” AND “bexagliflozin”  “Metformin” AND “ertugliflozin” |
| --- |

Appendix 1 shows the search words used in Pubmed to compile the articles referenced in this study comparing metformin monotherapy and GLP-1 RA/SGLT-2i.

**Appendix 2 Inclusion and Exclusion Criteria of Study Selection**

| **Inclusion Criteria:**  -Randomized Controlled Trials  -Patients with DMII  -One arm NOT taking MTF  -$\geq$12 weeks  -Outcome include surrogates for glucose lowering, macrovascular complications of DM, microvascular complications of DM, weight loss, safety | **Exclusion Criteria:**  -Letters, conference or case reports  -One arm trials  -Studies with insufficient data  -Non-human participants  -Use of combination products |
| --- | --- |

Appendix 2 includes the inclusion and exclusion criteria used in the search to compile the articles referenced in this study comparing metformin monotherapy and GLP-1 RA/SGLT-2i.
