# Supplementary material for: Narrative review of data supporting alternate first-line therapies over metformin in type 2 diabetes
Source: J Diabetes Metab Disord. 2024 Mar 25;23(1):385–94. doi: 10.1007/s40200-024-01406-6 (PMC11196467; doi:10.1007/s40200-024-01406-6)
Supplement: Supplementary file 2 — Supplementary Material 2 [file 40200_2024_1406_MOESM2_ESM.docx]

Dr. John Andraos, PharmD, BCACP, APh

Assistant Professor of Pharmacy

Western University of Health Sciences

309 E 2nd St

Pomona, CA 91766

626-363-3077

[jandraos@westernu.edu](mailto:jandraos@westernu.edu)

Editor-in-Chief

Journal of Diabetes and Metabolic Disorders

December 22, 2023

To whom it may concern,

We wish to submit an original research review article entitled “Review of Data Supporting Alternate First-Line Therapies Over Metformin in Type 2 Diabetes” for your consideration inside *Journal of Diabetes and Metabolic Disorders*.

We confirm that this work is original and has not been published elsewhere, nor is it under consideration for publication elsewhere.

In this paper, we review in depth research that made metformin a first-line agent and explore what literature supports the use of the more novel agents as first-line without metformin. SGLT-2i and GLP-1 RA are being used more and more frequently and have tremendous advantages over older agents used for diabetes. These newer drug classes lower cardiovascular disease, weight, and renal disease all while minimizing hypoglycemia. Because of these positive effects, recent guidelines have taken metformin off as the only first-line agent for diabetes and now say GLP-1 RA and SGLT-2i may be used preferentially. However, most of the positive effects of these drugs were proven when used with metformin. Our study does a comprehensive analysis of the literature supporting the historical use of metformin as first-line, the literature available analyzing the benefits of GLP-1 RAs and SGLT-2i without metformin’s use, and the literature available comparing these agents used as monotherapy versus metformin. Our article supports the change in guideline recommendation, but also reminds readers of the limitations of the research and utility of metformin in patients.

We confirm that we have no conflicts of interest to disclose.

Please address any correspondence concerning this manuscript to me at [jandraos@westernu.edu](mailto:jandraos@westernu.edu).

Thank you for your consideration.

Sincerely,

Dr. John Andraos, PharmD, APh

Assistant Professor of Pharmacy

Western University of Health Sciences
